# Genomic analysis of exceptional responder to regorafenib in treatment-refractory metastatic rectal cancer: a case report and review of the literature

**DOI:** 10.18632/oncotarget.18357

**Published:** 2017-06-03

**Authors:** Krittiya Korphaisarn, Jonathan M. Loree, Van Nguyen, Ryanne Coulson, Vijaykumar Holla, Beate C. Litzenburger, Ken Chen, Gordon B. Mills, Dipen M. Maru, Funda Meric-Bernstan, Kenna R. Mills Shaw, Scott Kopetz

**Affiliations:** ^1^ Department of Gastrointestinal Medical Oncology, The University of Texas MD Anderson Cancer Center, Houston, Texas, USA; ^2^ Department of Medicine, Division of Medical Oncology, Faculty of Medicine Siriraj Hospital, Bangkok, Thailand; ^3^ Department of Pharmacy, The University of Texas MD Anderson Cancer Center, Houston, Texas, USA; ^4^ Sheikh Khalifa Bin Zayed Al Nahyan Institute for Personalized Cancer Therapy (IPCT), The University of Texas MD Anderson Cancer Center, Houston, Texas, USA; ^5^ Department of Pathology, The University of Texas MD Anderson Cancer Center, Houston, Texas, USA

**Keywords:** metastatic colorectal cancer, regorafenib, survival, biomarker, response

## Abstract

We present the case of a 53-year-old male with metastatic rectal cancer who was treatment resistant to FOLFOX and FOLFOXIRI. Due to a Kirsten rat sarcoma viral oncogene homolog (*KRAS*) mutation, regorafenib was given in the third line setting. Surprisingly, the patient had a prolonged partial response that lasted 27 months. Mutational status was extensively evaluated to identify potential alterations that might play a role as predictive markers for this unusual event. A poorly characterized but nontransforming mutation in Fms-like tyrosine kinase 4 (*FLT4*) was present in the tumor. Prior to and at the time of clinical progression, we found amplification of fibroblast growth factor receptor 1 (*FGFR1) and* epidermal growth factor receptor *(EGFR)*, loss of the *FLT4* mutation, and gain of *KIT* proto-oncogene receptor tyrosine kinase *(KIT)* G961S suggesting potential roles in acquired resistance.

## INTRODUCTION

Regorafenib, an oral multikinase inhibitor, is used in treatment refractory metastatic colorectal cancer (mCRC) after failure of fluoropyrimidine, irinotecan, and oxaliplatin based therapies. In *RAS* wild type patients, progression following EGFR targeted therapy should also occur before use of regorafenib. The CORRECT clinical trial [1] demonstrated an overall survival (OS) benefit of regorafenib over placebo in treatment-refractory mCRC (6.4 months vs 5 months, Hazard ratio (HR) 0.77, 95% confidential interval (CI), 0.64-0.94, *P* = 0.0052). Mean duration of regorafenib treatment was 2.8 months with an objective response rate only being 1% (5/505). The benefit of regorafenib was also reported in Asian populations in the CONCUR trial [2], which demonstrated an extended OS in regorafenib treated patients compared to placebo (8.8 vs 6.3 months, HR = 0.55, 95%CI 0.44-0.77, *P* = 0.00016). However, there are no biomarkers predicting response to this drug and many patients suffer early progression during treatment with regorafenib. An extensive analysis of circulating tumor DNA and proteins from the CORRECT trial attempted to identify biomarkers able to predict response, however was unsuccessful [3]. Here, we report a case of an unusual deep and long-term response to regorafenib and present the molecular characterization of this patient to help elucidate potential determinants of this exceptional response.

## CASE REPORT

A 53-year-old male presented with lower abdominal pain, constipation, intermittent episodes of bright red blood per rectum, and significant weight loss of 20 pounds over 3 months. He had no significant past medical or family history, and physical examination was normal. The patient underwent a colonoscopy which demonstrated an exophytic mass in the rectum causing partial obstruction. Biopsy revealed moderate to poorly differentiated adenocarcinoma arising from a villous adenoma with high grade dysplasia. Staging investigations revealed liver limited multiple metastases, with the largest mass measuring 12 centimeters. Carcinoembryonic antigen (CEA) was within normal limit.

A 200 gene next generation sequencing (NGS) panel was performed on the biopsied primary and identified a *KRAS* mutation in codon G12S, a tumor protein p53 (*TP53*) mutation in codon R273C, an adenomatous polyposis coli (*APC*) mutation in codon R1450* and I742fs*, a protein phosphatase 1 regulatory subunit 3A (*PPP1R3A*) mutation in codon E271D, and a *FLT4*mutation, in codon F131S. *FLT4*, also known as vascular endothelial growth factor receptor 3 (VEGFR3)[4], is a member of the VEGFR family which can be targeted by regorafenib [5]. Since high VEGFR protein expression has been reported on colorectal cancer cells [6], we assessed the functional significance in the Ba/F3 cell reporter assay. This screen showed no IL-3 independent growth which is a surrogate for the transforming ability of this variant in *FLT4*. Molecular characterization of the tumor is shown in Table 1. CpG island methylator phenotype (CIMP) was high (abnormal methylation in 6/6 target genes) and microsatellite instability testing by immunohistochemistry demonstrated a microsatellite stable tumor.

**Table 1:** NGS panel results as assessed longitudinally throughout the course of a patient with an exceptional response to regorafenib

Gene	Diagnostic Biopsy (Pre-treatment)	Biopsy of Primary Tumor after Partial Response	Biopsy of Primary Tumor at Time of Discordant Response	ctDNA Sequencing at Time of Systemic Progression
Platform used	T200	T200.1	T200.1	Guardant 360
*KRAS* G12S	✓	✓	✓	✓
*TP53* R273C	✓	✓	✓	✓
*TP53* R175H	x	x	x	✓(minor alteration)
*APC* R1450*	✓	x	x	x
*APC* I742fs*	✓	✓	✓	✓ (minor alteration)
*FLT4* F131S	✓	x	x	x
*PPP1R3A* E271D	✓	x	x	x
*ATR* I774fs*	x	✓	x	x
*EP300* L1755V	x	x	✓	x
*WHSC1L1* E123Q	x	x	✓	x
*KIT* G691S	x	x	x	✓
*MYCL* amplification	x	✓ (7.4)*	✓ (2.5)*	x
*CDK4* amplification	x	✓ (3.1)*	✓ (2.7)*	✓ (2.4)*
*KRAS* amplification	x	✓ (3.1)*	x	✓ (2.4)*
*FGFR1* amplification	x	x	✓ (2.5)*	✓ (2.46)*
*EGFR* amplification	x	x	✓ (2.7)*	✓ (2.5)*
*MYC* amplification	x	x	x	✓ (2.49)*
*PIK3CA* amplification	x	x	x	✓ (2.58)*

*KRAS*;Kirsten rat sarcoma viral oncogene homolog, *TP53*; tumor protein p53, *APC*; adenomatous polyposis coli, *FT4*; Fms-Related Tyrosine Kinase, *PPP1R3A;* protein phosphatase 1 regulatory subunit 3A, ATR; ataxia telangiectasia and Rad3 related, *EP300*; E1A binding protein p300, *WHSC1L1*; Wolf-Hirschhorn Syndrome Candidate 1-Like 1, *KIT;* KIT proto-oncogene receptor tyrosine kinase, *MYCL*; v-myc avian myelocytomatosis viral oncogene lung carcinoma derived homolog, *CDK4*; cyclin dependent kinase 4, *FGFR1*; fibroblast growth factor receptor 1, *EGFR*; epidermal growth factor receptor, *PIK3CA*; phosphatidylinositol-4,5-bisphosphate 3-kinase catalytic subunit alpha, ctDNA; circulating tumor DNA, *copy number

**Figure 1:**
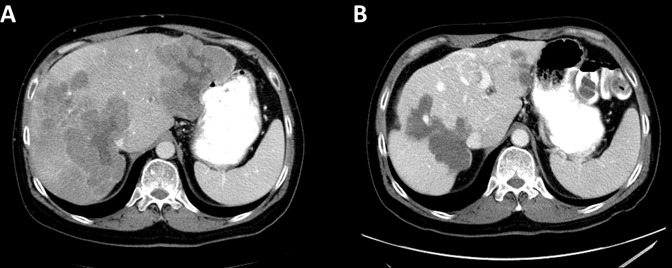
Abdominal CT with contrast (**A**) at baseline showed multiple liver masses, largest 9.8×9.5 cm. in the left lobe and 11.6×9.8cm in the right lobe (**B**) At best response, the liver masses were 5.4×4 cm in the left lobe and 8.3×9.5 cm in the right lobe.

Due to the patient's prior rectal bleeding and *in-situ* primary malignancy, FOLFOX was initiated with bevacizumab omitted. After 4 cycles of treatment, interval CT scan showed progression of the hepatic metastases and rectal mass according to the Response Evaluation Criteria in Solid Tumors (RECIST) version 1.1 guideline [7]. The patient's treatment was changed to FOLFIRINOX, with an initial partial response (PR) after 4 cycles. However, after 8 cycles the patient once again demonstrated progressive disease in the liver and rectum. The patient was subsequently started on regorafenib at a dose of 120 mg per day for 3 weeks each 28-day cycle as per MD Anderson's institutional dosing practice. Interval CT scan of abdomen after 2 months showed a dramatic response. Hepatic metastases decreased in size from 9.8 to 7.7 in the left lobe and 11.6 to 9.3 centimeters (cm) in the right lobe which was confirmed after 4 months. He continued on treatment without any dosing modifications. After 10 months of regorafenib, he required a dose reduction due to grade 2 hand-foot skin reaction (HFSR) which was most pronounced on the third week of each cycle. Subsequently, his dose was changed to 120 mg per day for the first two weeks and 80 mg per day for the third week. After 15 months of treatment, a flexible sigmoidoscopy was performed and showed an ulcerative non-obstructive mass at the site of the primary tumor which was biopsied and confirmed residual poorly differentiated adenocarcinoma. A repeat 200 gene NGS panel was performed on this biopsy and identified *KRAS* G12S, *TP53* R273C, and APC I742fs* which were previously reported at time of diagnosis. However, new gene alterations were identified in ataxia telangiectasia and Rad3 related (*ATR*) gene at codon I774fs*; and gene amplifications in v-myc avian myelocytomatosis viral oncogene lung carcinoma derived homolog (*MYCL*), cyclin dependent kinase 4 (*CDK4*)*,* and *KRAS*. Figure 1 showed the maximum response of the liver metastases after 17 months of regorafenib treatment.

Treatment with regorafenib was continued with good tolerance. After 20 months of regorafenib a CT scan of the abdomen showed stable liver metastases but increased size of the rectal mass. Re-biopsy of the rectal tumor was obtained to assess for mechanisms of resistance and sequencing identified *FGFR1* and *EGFR* gene amplifications; and an E1A binding protein p300 (*EP300*) mutation in codon L1755V, and a Wolf-Hirschhorn Syndrome Candidate 1-Like 1 (*WHSC1L1*) mutation in codon E123Q. Concurrent chemo-radiation (CCRT) therapy with capecitabine 650mg/m2 twice daily with a total of 50.4Gy was initiated and regorafenib was placed on hold. Upon completion of CCRT, regorafenib was re-initiated with continued disease control in the liver. Unfortunately, after 27 months of regorafenib treatment, an abdominal CT revealed progression of the liver metastases. Re-biopsy of the liver was attempted but there were no viable cells to characterize. Therefore, circulating tumor DNA (ctDNA) sequencing was used to characterize alterations after regorafenib progression. Analysis revealed a mutation in *KIT* at codon G961S, *PIK3CA* and *MYC* gene amplifications that were not noted on prior testing and confirmed *FGFR1* and *EGFR* amplifications which were previously identified in the progressed rectal tumor tissue. The mutational profile is summarized in Table 1 and Supplementary Table 1.

As the patient had not received any other prior anti-VEGF therapy, he was started on irinotecan plus aflibercept. Restaging CT scans after 2 and 4 months showed stable disease, however the patient developed grade III diarrhea during therapy leading to the omission of subsequent irinotecan after 4 months. The patient continued aflibercept for a further 2 months at which point he was found to have hepatic progression. The patient was subsequently transitioned to best supportive care.

## DISCUSSION

We report the case of an exceptional responder to regorafenib in mCRC and describe the alterations identified through molecular testing, anticipating to elucidate a potential mechanism of sensitivity in this patient.

Regorafenib, an oral mutikinase inhibitor, can inhibit activity of several protein kinases, including those involved in tumor proliferation (*KIT, PDGFR* and *RET*), tumor angiogenesis (*VEGFR1-3, TIE2*), and tumor microenvironment (*PDGFR-B, FGFR*) [5, 8, 9]. The Food and Drug Administration approved regorafenib in 2012 for the treatment of mCRC after failure of standard therapies, including fluoropyrimidine, oxaliplatin, and irinotecan-based chemotherapy, anti-VEGR therapy, and anti-EGFR therapy in *KRAS* wild type tumors. Regorafenib showed benefit in both *KRAS*-wild-type and *KRAS*-mutant subgroups [1, 2].

Prior attempts to identify a useful biomarker to select patients who will benefit from regorafenib have assessed stereotypic mCRC aberrant genes including *KRAS*, *BRAF*, *PIK3CA*, and MMR status and failed to correlate mutations in any of these genes to treatment response [2, 3]. Teufel et al suggested a benefit of regorafenib therapy in patients with high-risk molecular characteristics defined by gene expression clusters (HR = 0.10; 95%CI 0.02 - 0.35) compared to a lower-risk subgroup (HR = 0.58; 95%CI 0.44 - 0.77) although this has not yet been validated [3]. Moreover, markers of angiogenesis may have potential utility in identifying responders. Eisen et al reported higher baseline TIMP metallopeptidase inhibitor 2 (TIMP2) and soluble tyrosine kinase with immunoglobulin like and EGF like domains 1 (TIE1) which were correlated with regorafenib treatment response [10]. Data from CORRECT [3] also demonstrated that high levels of soluble protein TIE1 were associated with OS benefit in the regorafenib group. Additionally, Giampieri et al reported that patients who harbored VEGF-A rs2010963 germline polymorphism showed better PFS (HR = 0.49, 95%CI 0.33-0.81) and OS (HR 0.52, 95%CI 0.34-0.99) when treated with regorafenib compared to those without these polymorphisms [8]. While hypothesis generating, all of these angiogenic markers suffer from limited power due to multiple comparison and require further studies.

The patient reported here had an exceptional response to regorafenib of 27 months, which has never been reported previously. The most recent published data from Japan [11] reported 18 months of partial response in a patient with mCRC treated after progression on FOLFOX, FOLFIRI, and XELOX regimens. However, they did not report any molecular analysis. In this study, we utilized sequential molecular testing before, during, and upon progression of regorafenib treatment. We found several gene mutations, including *KRAS codon G12S, TP53 codon R273C,* and *APC* codon I742fs*which persisted from diagnosis through treatments. We also found several transient mutations that occurred during regorafenib treatment including *APC* codon R1450*, PPP1R3A codon E271D, *ATR* gene in codon I774fs*, *EP300* codon L1755V, and *WHSC1L1* codon E123Q. However, these genes do not have biologic rationale to support their use as a predictive biomarker, and instead likely reflect clonal diversity over time.

*FLT4* mutations are rare in CRC and have been reported in only 2.4 % (5/212) in sequenced CRC in the Cancer Genomic Atlas (TCGA) dataset [12, 13]. *FLT4* F131S located within the extracellular region of FLT4 protein [14]. Previously *FLT4* F131S has not been functionally characterized; therefore this mutation was functionally analyzed in the Ba/F3 system which revealed that this mutation does not induce growth factor independent cell growth and thus is characterized as likely non-transforming/benign. Nevertheless, it cannot be ruled out that *FLT4* mutations might sensitize cells/tumors to regorafenib treatment. Further experiments that characterize this mutation with regards to its therapeutic effect might be needed.

The mechanisms of pre-existing and acquired resistance to regorafenib are unknown. Recent data from a pre-clinical study demonstrated Notch-I upregulation in regorafenib resistant tumor cells and inhibition of Notch-I in resistant cells partially restored sensitivity to the regorafenib treatment *in vitro*. These results suggest Notch as potential mechanism of acquired resistance [15]. Gene amplifications are a common mechanism of acquired resistance to targeted therapies in CRC. Examples include *BRAF* gene amplification in MEK inhibitor treated tumor [16] , *HER2* and *MET* amplification in anti-EGFRab treated tumor [17, 18] . Many acquired gene amplifications were identified in the patient's tumor profile (Table 1); several of these amplifications were present in the responding tumor (*MLCL, CDK4, and KRAS*) and are less likely to be associated with resistance. Others such as *MYC* and *PIK3CA* were only present in the cfDNA but not seen in the progressing rectal primary. In contrast *FGFR1* and *EGFR* were present at the time of progression of the rectal primary and later upon progression of the liver metastases, and are therefore candidate resistance mechanism.

*FGFR1* is a gene that encodes a member of FGFR family which includes four receptor tyrosine kinases, *FGFR1-4* [19]. *FGFR1* gene amplification has been reported in numerous malignancies including breast cancer and squamous cell carcinoma of lung cancer, head and neck cancer, and esophageal cancer [20–23]. *FGFR1* amplifications have been reported in 2.8% (6 cases) of 212 sequenced CRC in the TCGA dataset [12, 13]. Although *FGFR1* has recently emerged as a promising target in non-small cell lung cancer, data from CRC are limited. EGFR belongs to a family of cell signaling receptors and is known to activate a cascade of multiple signaling pathways. The presence of an EGFR abnormality; including mutation, amplification, and overexpression, can result in over activity of EGFR protein and excessive proliferation [24]. *EGFR* amplifications have been reported in 0.5% (1 case) of 212 sequenced CRC in the TCGA dataset [12, 13]. Although EGFR mutations have been reported to predict sensitivity to EGFR tyrosine kinase inhibitors in lung cancer [25], little is known about the impact of *EGFR* amplifications in either for selecting patient to anti-EGFR treatment or as a role in resistance.

Each of the above amplifications were noted in pathways that are adjacent or in line with a pathway targeted by regorafenib and our molecular characterization shows multiple concurrent potential resistance mechanisms induced by regorafenib. However, to our knowledge, no gene amplification has previously been established as a potential resistance mechanism for regorafenib.

*KIT* was the only mutation noted upon tumor progression during regorafenib. *KIT* encodes the human homolog of the proto-oncogene c-kit that belongs to the type III tyrosine kinase receptor family [26]. Binding of its endogenous ligand, stem cell factor (SCF) initiates multiple downstream signaling pathways [27–29]; including mitogen-activated protein kinase (MAPK) pathway, phosphatidylinositol 3-kinase (PI3K)/AKT pathway, Janus kinase/signal transducers and activators of transcription (JAK/STAT) pathway, PLC- γ signaling transduction pathway and Src kinase signaling transduction pathway, leading to cell proliferation, survival and migration. *KIT* mutations have been reported in 2.8% (6/212) of sequenced CRC in the TCGA dataset [12, 13]. *KIT* G961S alteration has not been functionally characterized. It is located at the C-terminal end of the protein, outside of any known function domain [14]. Although, *KIT* G916S has not been reported with any clinical significance, the acquisition of any mutation in a kinase targeted by regorafenib suggests that *KIT* G961S might play a role in acquired resistance.

## CONCLUSION

We report a case with an unusually prolonged response to regorafenib in mCRC and we highlight the development of *FGFR1*/*EGFR* amplifications and a *KIT* G961S mutation as potential mechanisms of acquired resistance in this patient. The molecular features of this exceptional responder may provide insight into genomic alterations that develop during regorafenib treatment which may lead to acquired resistance.

## APPENDICES

### Methods

#### T200 gene panel

The T200 is a next generation sequencing panel that provides sequencing coverage of all exons for 201 cancer related genes. The panel consists of 4874 exons encoding 938607 bases and was designed with a higher read depth in order to provide the ability to call mutations at lower allele frequencies (down to 1%). Detailed methods associated with this assay have been previously published [30].

#### Guardant 360TM assays

The Guardant 360TM is a commercially available next generation sequencing panel developed for use with circulating tumor DNA (ctDNA). The panel consists of 68 cancer related genes and is able to identify mutations and copy number alterations. Cell free DNA is extracted from plasma and genomic alterations are analyzed by massively parallel sequencing of amplified target genes. The minimum detectable mutant allele is dependent on the concentration of ctDNA in a patient's serum at the time of blood draw [31].

#### CIMP methylation

Assay is performed using either formalin-fixed, paraffin-embedded tissue blocks or frozen tissue samples. DNA extracted from formalin-fixed, paraffin-embedded tissue or frozen tissue samples is treated with bisulfite to convert unmethylated cytosine to uracil. PCR amplification of both unmethylated and methylated MINT1, MINT2 and MINT31 loci, and promoter sequences of p14, p16 and hMLH1 genes is performed and methylation status is assessed by pyrosequencing. The tumor is considered CIMP High, if at least 40% of markers tested show methylation, and CIMP Low if < 40% markers show methylation.

#### IL3 dependency Ba/F3 assay

An IL-3 dependent murine Ba/F3 cell reporter model was used to evaluate the functional impact of a FLT4 F131S mutation. The procedure was same as described previously [32] with few modifications. Briefly, this pro-B cell line is dependent on IL-3 for proliferation. Oncogenic transformation with a mutation results in IL-3 independent growth, thus highlighting a functionally significant mutation. Ba/F3 cells were introduced with FLT4 F131S mutant using lentivirus approach and incubated in medium with 0.5 pg/ml IL-3 which is 0.01% of regular IL-3 concentration used in cell line maintenance. Trace amount of IL-3 in medium delays IL-3 depletion-mediated cell death and gives time to the cells to adapt oncogenic mutant. Cell viability was measured after 1, 1.5, and 2 weeks.

## SUPPLEMENTARY MATERIALS FIGURES AND TABLES


