# Supplementary material for: Genomic analysis of exceptional responder to regorafenib in treatment-refractory metastatic rectal cancer: a case report and review of the literature
Source: Oncotarget. 2017 Jun 3;8(34):57882–8. doi: 10.18632/oncotarget.18357 (PMC5593691; doi:10.18632/oncotarget.18357)
Supplement: Supplementary file 1 [file oncotarget-08-57882-s001.pdf]

Genomic analysis of exceptional responder to regorafenib in treatment-refractory metastatic rectal cancer: a case report and review of the literature

SUPPLEMENTARY MATERIALS

Supplementary Table1: NGS panel results including full details of mutations noted in Table 1 from body of manuscript in a patient with an exceptional response to regorafenib.

	Platform	Gene	Allele frequency (%)	Chromosome	Standardized Nomenclature (HGVS)	Location	DNA change	Protein change	dbSNP ID	COSMIC ID
**Diagnostic biopsy**	T200	KRAS	34.72	12	NM_033360.3(KRAS):c.34G>A p.G12S	Exon 2	SNV	Missense	rs121913530	COSM517
		TP53	38.44	17	NM_000546.5(TP53):c.817C>T p.R273C	Exon 8	SNV	Missense	rs121913343	COSM10659
		APC	12.04	5	NM_000038.5(APC):c.4348C>T p.R1450*	Exon 15	SNV	Nonsense	rs121913332	COSM13127
		APC	44.8	5	NM_001127511.2(APC):c.2224delA p.I742fs*	Exon 15	SNV	Frameshift	NA	NA
		FLT4	21.38	5	NM_182925(FLT4):c392T>C p. F131S	Exon 3	SNV	Missense	NA	NA
		PPP1R3A	5.33	7	NM_002711(PPP1R3A):c813A>C p.E271D	Exon 2	SNV	Missense	NA	NA
**During treatment**	T200.1	KRAS	50	12	NM_004985.3(KRAS):c.34G>A p.G12S	Exon 2	SNV	Missense	rs121913530	COSM517
		TP53	76.92	17	NM_001126116.1(TP53):c.817C>T p.R273C	Exon 8	SNV	Missense	rs121913343	COSM10659
		APC	53.16	5	NM_001127511.2(APC):c.2224delA p.I742fs*	Exon 15	SNV	Frameshift	NA	NA
		ATR	11.59	3	NM_001184.3(ATR):c.2320delA p.I774fs*	Exon 10	SNV	Frameshift	NA	COSM214499
**After progression**	T200.1	KRAS	22.76	12	NM_004985.3(KRAS):c.34G>A p.G12S	Exon 2	SNV	Missense	rs121913530	COSM517
		TP53	28.69	17	NM_001126116.1(TP53):c.817C>T p.R273C	Exon 8	SNV	Missense	rs121913343	COSM10659
		APC	28.63	5	NM_001127511.2(APC):c.2224delA p.I742fs*	Exon 15	SNV	Frameshift	NA	NA
		EP300	16.74	22	NM_001429.3(EP300):c5263c>G p.L1755V	Exon 31	SNV	Missense	NA	NA
		WHSC1L1	15.08	8	NM_023034.1(WHSC1L1):c.367G>C p.E123Q	Exon 2	SNV	Missense	NA	NA
	Guardant 360	Major alterations								
		KRAS	13.73	12	NM_033360.2(KRAS):c.34G>A p.G12S	Exon 2	SNV	Missense	rs121913530	COSM517
		TP53	27.94	17	NM_000546.5(TP53):c.817C>T p.R273C	Exon 8	SNV	Missense	rs121913343	COSM10659
		KIT	2.97	4	NM_000222.2:c.2881G>A p.G961S		SNV	Missense	rs773828910	NA
		Minor alteration								
		TP53	0.26	17	NM_000546.5(TP53):c.524G>A p.R175H	Exon5	SNV	Missense	rs28934578	COSM10648
		APC	0.70	5	NM_001127511.2(APC):c.2224delA p.I742fs*	Exon 15	SNV	Frameshift	NA	NA

KRAS;Kirsten rat sarcoma viral oncogene homolog, *TP53*; tumor protein p53, *APC*; adenomatous polyposis coli, *FT4*; Fms-Related Tyrosine Kinase, *PPP1R3A;* protein phosphatase 1 regulatory subunit 3A, ATR; ataxia telangiectasia and Rad3 related, *EP300*; E1A binding protein p300, *WHSC1L1*; Wolf-Hirschhorn Syndrome Candidate 1-Like 1, *KIT;* KIT proto-oncogene receptor tyrosine kinase
